# Perspectives of patients, family members, and health care providers on late diagnosis of breast cancer in Ethiopia: A qualitative study

**DOI:** 10.1371/journal.pone.0220769

**Published:** 2019-08-01

**Authors:** Alem Gebremariam, Adamu Addissie, Alemayehu Worku, Mathewos Assefa, Eva Johanna Kantelhardt, Ahmedin Jemal

**Affiliations:** 1 Department of Public Health, College of Medicine and Health Sciences, Adigrat University, Adigrat, Ethiopia; 2 School of Public Health, College of Health Sciences, Addis Ababa University, Addis Ababa, Ethiopia; 3 Radiotherapy Center, School of Medicine, Addis Ababa University, Addis Ababa, Ethiopia; 4 Institute of Medical Epidemiology, Biostatistics and Informatics, Martin-Luther-University, Halle, Germany; 5 Surveillance and Health Services Research, American Cancer Society, Atlanta, Georgia, United States of America; Emory University School of Public Health, UNITED STATES

## Abstract

**Background:**

Most women with breast cancer in Ethiopia are diagnosed at an advanced stage of the disease, but the reasons for this have not been systematically investigated. This study, therefore, aimed to explore the main reasons for diagnosis of advanced stage breast cancer from the perspective of patients, family members, and health care providers.

**Methods:**

A qualitative study with in-depth interviews was conducted with 23 selected participants at Tikur Anbessa Specialized Hospital, Oncology Clinic using a semi-structured interview guide. These participants were 13 breast cancer patients, 5 family members, and 5 health care providers. Data were transcribed into English, coded and analyzed using thematic analysis.

**Results:**

Awareness about the causes, risk, initial symptoms, early detection methods, and treatment of breast cancer were uncommon, and misconceptions about the disease prevailed among breast cancer patients and family members. There was a sense of hopelessness and uncertainty about the effectiveness of conventional medicine amongst patients and family members. Consequently, performing spiritual acts (using holy water) or seeking care from traditional healers recurred amongst the interviewees. Not taking initial symptoms of breast cancer seriously by the patients, reliance on traditional medicines, competing priorities, financial hardship, older age, fear of diagnosis of cancer, and weak health systems (e.g., delay in referral and long waiting period for consultation) were noted as the main contributors to late diagnosis. In contrast, persuasion by family members and friends, higher educational attainment, and prior experience of neighboring women with breast cancer were mentioned to be facilitators of early diagnosis of breast cancer.

**Conclusions:**

The causes of late diagnosis of breast cancer in Ethiopia are multi-factorial and include individual, cultural, and health system factors. Interventions targeting these factors could alleviate the misconceptions and knowledge gap about breast cancer in the community, and shorten waiting time between symptom recognition and diagnosis of breast cancer.

## Introduction

Cancer is becoming a major public health problem in most economically developing countries, including Ethiopia [1]. In Ethiopia, breast cancer is the most commonly diagnosed cancer and the leading cause of cancer death in women [2], accounting for about 32% of the total cases [3] and 19.4% of all cancer deaths [4]. Further, over two-thirds of breast cancers in Ethiopia are diagnosed at late stages of the disease [5]. In addition to the late stage at diagnosis, access to evidence-based treatment is limited in Ethiopia, resulting in poor survival [5, 6].

A previous systematic review conducted by Espina and his colleagues has summarized the reasons for delayed diagnosis of breast cancer patients in Africa. The main reasons noted by this study were low educational attainment, lack of awareness on breast cancer and its detection methods, type of initial symptoms, fear of the disease or its treatment, traditional medicine use, financial difficulties, lack of access to healthcare, number and type of health care providers contacted before diagnosis, delayed referrals or non-referrals, misdiagnosis, wrong advice or false reassurances, and delays in obtaining diagnostic confirmation [7]. This review was mainly based on quantitative studies, and it may not have captured information that could be elucidated from qualitative studies through probing of participants [8]. In addition, the studies included were all based on data obtained before 2014. Since then, various changes have occurred within Ethiopia and Africa such as more political attention to cancer care [9], but late stage disease presentation is still a major problem [10]. The Ethiopian National Cancer Control Plan was drafted in 2015 for the period of 2016–2020 aimed at improving early diagnosis of cervical and breast cancer. For cervical cancer, the government has purchased hundreds of cryotherapies to implement visual inspection with acetic acid in major hospitals in the country. For breast cancer, the government plans to promote early detection of symptomatic breast cancer patients and screening asymptomatic high risk women through promoting breast self-awareness, and clinical breast examination for all women coming to health institutions for other conditions [11]. In addition, the government is constructing five regional cancer treatment centers, subsidizing the procurement of lifesaving chemotherapy for all public facilities, training more health professionals (oncology specialists, nurses and pharmacists), and collaborating with different partners to build the capacity of the health care providers.

Understanding the recent specific barriers to early diagnosis that Ethiopian breast cancer patients are facing is important for planning targeted interventions that help to mitigate premature death of women from breast cancer [12]. Initial exploration using qualitative research methods were considered most appropriate because these methods allow for an in-depth understanding of opinions, thoughts and feelings of respondents, and obtaining more contextualized information on the barriers encountered by the respondents [13]. However, there are few qualitative studies which used first-hand patient narratives to explore the specific barriers to early detection of breast cancer among breast cancer patients in Ethiopia. Dye and his colleagues, using mixed methods, have investigated the experiences of initial symptoms and actions [14], patient navigation [15] and beliefs and practices [16] of breast cancer in Ethiopia. These studies have documented that a substantial proportion of women with breast cancer first seek care in the form of traditional medicine, waited for about one and half year before medical consultation, and face difficulties in navigating the healthcare system for timely diagnosis of their cancer. However, these results were based on data collected approximately 10 years ago and were limited to the beliefs and practices of patients. Hence, the evidence from these studies may not reflect the current causes of late diagnosis of breast cancer in the country in view of the rising burden of the disease and changing healthcare system.

In this study, we investigated both patient and health system-related reasons for late diagnosis of breast cancer from the patients’, family members’, and health care providers’ perspectives in Addis Ababa. The information obtained from this study will help identify gaps and barriers to early diagnosis. It also provides fact-based material to develop “stories” that illustrate the compelling need of improved care.

## Methods

The study was conducted at Tikur Anbessa Specialized Hospital (TASH) Oncology Center. TASH is a teaching hospital of the College of Health Sciences of Addis Ababa University, and tertiary referral hospital for the country. The hospital has a separate building for cancer treatment (oncology clinic). This hospital was chosen because it is the referral hospital for cancer care and hosts the only radiotherapy machine in the country.

### Study design

A phenomenological study was used to explore the barriers for early diagnosis of breast cancer. Phenomenological method is useful in studying participants’ lived experiences of a phenomenon, which in this case is the experience of breast cancer diagnosis, from patients, family members and health care providers’ perspective [17]. This study was conducted as a baseline to a broader ongoing follow-up study in Addis Ababa documenting the experiences of women with breast cancer from recognition of symptoms to diagnosis, treatment, and survivorship, and end of life [18].

### Recruitment of study participants

A total of 23 In-depth Interviews (IDI) were conducted with 13 breast cancer patients, 5 family members, and 5 health care providers. Participants were recruited through purposeful sampling [19]. Women aged 18 years and above who were diagnosed with breast cancer in 2016 and treated at the oncology clinic from March–July 2017, were included in the study. Similarly, family members aged 18 years and above who accompanied breast cancer patients were included in the study. Health care providers who had a minimum of bachelor degree in health science, and have been providing care to cancer patients at the oncology clinic for more than a year were included in the study. The sample size was determined based on information saturation; recurrent patterns became evident in the participants’ narrations [20].

### Interviews

Eligible participants were identified with the help of the head nurse of the oncology clinic. After we obtained willingness to participate, an appropriate place and time for an interview was arranged. Interviews were conducted face to face with participants individually in a private room which was either in the cancer registry office or head nurse’s office of the oncology clinic. The primary investigator (AG) and three public health experts who had a Master in Public Health and experienced in qualitative research conducted the in-depth interviews. The four interviewers (2 males and 2 females) were paired into two groups consisting one note taker and one interviewer. None of the interviewers worked at the oncology clinic, therefore, the interviewers likely had no direct influence on the interview responses.

A semi-structured interview guides were used to interview the three groups of respondents: breast cancer patients, family members and health care providers. Different interview guides were used for the three target groups (S1 File). The interview guides consisted of open-ended questions. For instance, some of the questions asked to the breast cancer patients were: “What is known about breast cancer in the community? Have you ever heard of breast cancer before you recognized the first symptoms of your illness? Tell me about your experience with breast cancer from the first symptoms recognition to diagnosis? What are the major challenges you have faced during diagnosis? Why do breast cancer patients seek medical care late? (What affected that), and What measures do you suggest to make sure breast cancer diagnosis happens early?” In addition to these main questions, the interview guide included probing questions.

Interviewers guided the respondents during the interview until all of the questions listed in the interview guide were inquired. Probing was used to inquire further explanation of the responses of the participants. All of the interviews were audio-recorded. Field notes were recorded during the in-depth interview to include keynotes, observable non-verbal cues of the participants. Each of the interviews took 40 minutes on average.

### Data analysis

The transferability of the findings was ensured by using person triangulation [19]: data were collected from three groups of participants (patients, family members and health care providers) to triangulate and validate the findings. This helped to generate a more comprehensive data. The data collectors and investigators were debriefed on a daily basis to discuss themes and issues for exploration, and to verify saturation of information. Audio data were transcribed verbatim into Microsoft Word files. All transcripts of the interviews were checked for errors by simultaneous reading of the transcripts with the audio-recorded voices. Each transcript and corresponding field notes were read thoroughly to gain a sense of the respondent’s experience as a whole. Verbatim transcripts of the data were imported into NVivo software version 11 for computer-based data coding and reduction without compromising the central idea. Line-by-line coding was then conducted by the primary investigator (AG). Codes were compared based on their differences and similarities, and sorted into categories. Categories then were grouped into themes, which were analyzed using thematic analysis [21]. Quotes that best described the various categories and expressed what was said frequently in several groups were chosen, and presented in italics.

### Ethical considerations

The study was approved by Institution of Review Board of Addis Ababa University College of Health Science (ref: 018/17/SPH) and participants provided fully informed consent to participate by reading a participant information sheet and signing a consent form.

All documents were kept private and confidential. All audio-recorded interviews were reviewed by the transcriber and the principal investigator only, and each participant was identified by specific code number, rather than by name.

## Results

A total of 23 in-depth interviews were conducted with breast cancer patients, family members, and health care providers of Tikur Anbessa Specialized Hospital Oncology clinic (Table 1).

**Table 1:** Study participants’ characteristics and code, TASH, Oncology Clinic, Addis Ababa, Ethiopia, 2017.

Socio-demographic Characteristics	Breast cancer patients (P01-13)	Family Members (R01-05)	Health Care Providers (HP01-05)
Participants age* **(years)**			
<40	7	3	-
40–50	5	1	-
>50	1	1	-
**Highest level of education**			
Not attended school	7		
Primary school (1-8^th^)	5	1	
Secondary school (9-12^th^)	2	1	
Diploma and above	1	2	
BSc. Degree		1	3
MSc. Degree			2
**Occupation**			
Housewife (homemaker)	2		
Employed outside the home	8	3	
No job	3	2	
Health care providers			5

P: breast cancer patient; R: Relatives; HP: Health Care Providers BSc.: Bachelor of Science, Master of Science

*the age of health care providers is omitted to keep anonymity

Based on thematic analysis, the responses of the participants revealed eight common reasons for late diagnosis, and three factors contributing to early diagnosis.

### Reasons for late diagnosis of breast cancer

#### Lack of awareness about breast cancer in the community

The study participants revealed a low level of awareness of breast cancer. Before they recognized the first breast cancer symptoms, breast cancer-related discussions and screening were uncommon. Almost all of the breast cancer patients had never heard of breast cancer before they sought treatment for their illness.

*“Before I recognized the first symptom of my illness*, *I have never heard of breast cancer*. *Rather*, *I heard women say I have pain in my breast…” (P08)*

*“… People in my community are not aware of breast cancer or they have wrong information about cancer*…*” (HP04)*

#### Disregarding or misattribution of breast cancer symptoms

The majority of the patients noticed a painless breast lump or swelling accidentally. The painless nature of the lump made the patients disregard its potential severity, and they usually attributed it to non-cancer illness, mainly sun stroke, locally known as “mitch”. One breast cancer patient who initially noticed a painless lump waited for about a year without seeking any medical care. Once she noticed her nipple inverted, she worried and sought medical care.

*“I thought it was just a disease not as severe as this… I thought it was caused by the smell from the place I worked at… I thought it was something related with mitch* …*” (P02)*

*“I noticed a small swelling in my breast*. *But I was not worried about it*. *It started to extend its tail to my arm pit*. *Then I went to the health center*. *They told me to go to the hospital… I did not trust them*. *I considered the disease as nothing*. *When I felt severe pain*, *I returned back to the healthcare facility*. *The doctor became visibly angry at me*.*” (P01)*

#### Misperceptions about breast cancer treatment and its outcomes

The study participants noted the presence of delusions about the causes, treatment and outcomes of cancer among the patients and their community. Amongst interviewees (patients and family members), many had no response to the question *“what do you think is the cause of breast cancer*?*”* However, they speculated on the causes of breast cancer based on their own perspectives and their communities. The speculated causes include sun stroke (M*itch*), supernatural force as a punishment for their sins, evil eye, use of contraceptive, blunt trauma to their breast, failure to breastfeed, poor breast care, inheritance, and climate change. “*Mitch*” was a recurrent speculated cause of their illness, as some believe it to be related to prolonged exposure to sunlight, especially during baking and preparing food at open field (out of the home or shelter), and prolonged exposure to food odor during cooking.

*“… Mine is caused by mitch because of excessive sun exposure*. *It happened when I took off my shirt during the hottest time of the day while working as a street vendor*.*” (P02)*

*“… They (women with breast cancer) feel like they did sinful things to deserve such disease*. *Some of the patients relate the disease with the things they eat and drink or smoke…” (HP03)*

A common view amongst interviewees was that cancer is a severe, deadly and incurable disease. There was a sense of hopelessness and fear of death from cancer amongst interviewees (patients and family members). These views were expressed mainly due to the doubt they had on the effectiveness of the conventional treatment. Such views were prevailing in those who were considered aware of cancer and those who had a bad experience.

*“How am I going to forget about the disease*? *I am carrying it… I think that I am going to die… other women also think that the disease cannot be cured and the person who has it is just waiting to die*.*” (P09)*

*“…I told you that people think cancer as a fatal disease …That was how my mom felt*. *She tried to give us everything and did not even think about herself at all*. *She perceives as she does not have life after the incidence*.*” (R03)*

*“… Some patients totally give up and refused to take the treatment*. *They preferred to go to traditional places/ holy water…” (HP05)*

#### Non-medical management of breast cancer symptoms

Performing spiritual acts (such as using holy water) or seeking care from traditional healers was common amongst the breast cancer patients and family members interviewed; this was commonly the primary response to the first symptoms. Medical consultation was often only then considered if the patients did not consider these remedies successful. It was not unusual for subjects ignore the medical advice received, and return home to use different traditional medicines.

*“… I went to a traditional healer and he provided me an ointment*, *which I used for about 4 months*. *Then there was no change with the swelling… I waited for more than a year without visiting health facility …” (P12)*

*“… There were people who completely healed by holy water… While I was in the hospital*, *one of my relatives advised me not to get breast surgery and rather to go to a traditional healer (Name)*. *But I refused her advice …” (P06)*

*“… There is traditional medicine even in Addis Ababa (capital city)*. *… They insert something to the affected breast using syringe*. *They say that the medicine will get rid of the cancer from the breast* …*” (P02)*

#### Fear of cancer diagnosis

Though issues related to fear of diagnosis were not particularly prominent in the interviewees, few reported fearing the confirmation of their diagnosis. This fear was mainly attributed to what the participants had heard in the community about the severity of cancer. A common view among interviewees was that in their community the word cancer is dreaded as incurable and fatal, and that cancer is difficult to talk about because of this perception.

*“I do not know. When I recognized my first symptoms*, *I thought I will die of the disease immediately if it turns out to be cancer. I was afraid of going to the health facility … I lost my hope because in the community, cancer is known as an incurable disease. It is called a killer.” (P02)*

Almost all of the in-depth interviewees (patients and family members) revealed that confirmation of breast cancer was a shocking and scaring news for the clients and their families. This was due to low suspicion of breast symptoms as a sign of cancer, perceived seriousness of cancer, uncertainty about effectiveness of conventional treatments, and changes in body image after surgical removal of breast cancer. The interviewees explained their feelings on mastectomy as follow:

*“Oh, my God, I felt many things, having a breast removal at old age … I had no pain at all*. *I felt why I have to go through breast removal at this age. I should die and get buried with dignity. I felt very sad.” (P13)*

*“… They say that it is shameful if someone hears about it … A person should die with full body parts*, *but part of the body will be lost due to the breast removal*.*” (R03)*

*“… He (physician) told me that he has to cut out my whole breast; and I was shocked and cried*.*” (P06)*

#### Competing priorities

Multiple interviewees responded that family responsibilities, such as looking after children, and the inability to take time off from work were their reasons to pay little attention to their first symptoms, usually a painless lump, of the breast illness.

*“… We have children who should go to the school; we need to prepare food… We have a lot of work… You cannot leave them and run to the health facility*.*” (P05)*

#### Financial insecurity

The cost of transportation, investigation and treatment was also mentioned as a reason for late diagnosis. There were patients who reported returning home after being referred to a higher health facility for financial reasons, while others reported selling their property or borrowing money to get medical care.

*“… I went to the health center*. *They told me to go to the hospital*, *but I did not have money to go to the hospital*. *Instead*, *I went to my home and stayed for months*.*” (P01)*

“*… We had no money*. *We were forced to sell our cattle and our crops to cover the medical cost…” (P05)*

“… *I thought the cost of diagnosis was cheap*, *but it cost me 7500 birrs … I borrowed money from different sources as I did not want to see my wife die*.*” (R01)*

#### Health system-related barriers

Physicians misunderstanding of the first symptoms (telling patients not to worry about their symptoms), inappropriate reassurance as the lump is benign without biopsy, long waiting times to receive diagnostic confirmation, few diagnostic centers, patient load, and poor provider-patient communication and counseling were all reported as reasons for late diagnosis of breast cancer.

*“… I noticed something a small lump on my breast* … *I woke up in the morning and went to my doctor*. *He told me it could be a tumor*. *I asked him if it could be a cancer because I heard about it on TV*. *He told me it is not a cancer*.*” (P07)*

*“… There is long waiting time to get treatment*. *As a result*, *many people are at higher risk of death …” (HP01)*

### Facilitators of early diagnosis of breast cancer

#### Persuasion by family members and friends

We found that family members and friends had a positive impact on the decision of patients to seek medical care.

*“… The influence of my friend working in the hospital made me seek medical care* … *Had she did not push me*, *I would not have sought medical care*. *…” (P06)*

#### Prior knowledge of someone with breast cancer

Knowing someone who was treated for breast cancer was reported to be a facilitator of early medical consultations.

*“… I know a mother who was treated for breast cancer*. *When I felt lump in my breast*, *I was shocked and immediately went to the hospital* …*”* ***(****P04)*

#### The literacy level of the women

Interviewees felt that women at a younger age with higher literacy level were more likely to be early health care seekers compared to older or less literate women. These views expressed mainly in relation to the role of education on women’s interpretation of the first symptoms of breast cancer and decision to seek medical care.

*“Younger women are better in seeking care*. *Older women did know nothing…” (P08)*

*“Those who are educated are more likely to seek care earlier*… *It is due to lack of knowledge…” (P05)*

### Participants’ suggestions to mitigate late diagnosis

In all cases, the informants suggested public health campaigns and programs to enhance awareness about breast cancer symptoms, signs, and benefit of early identification and treatment as an approach to mitigate the rising burden of the disease in the country. Involving breast cancer survivors in breast health education as community educators was also suggested.

*“… We should use breast cancer survivors to educate the community that cancer can be cured if patients seek medical care as soon as they noticed the symptoms… I can teach the community about what I experienced with the breast cancer*. *I do not think any one of the breast cancer survivors will disagree to educate the community and save the lives of other women*.*” (P03)*

The participants emphasized the importance of regular breast self-examination and seeking medical attention immediately following recognition of abnormal breast symptoms. The participants also suggested that interventions by the healthcare system to establish and expand counseling services, to reduce waiting times for diagnosis and treatment (expand diagnostic and treatment centers), and to mitigate the financial burden of cancer in patients and family members.

*“… The burden of the disease is increasing; most of the services*, *however*, *are given in Tikur Anbessa Specialized Hospital only*. *Therefore*, *the government should open more facilities providing such services and strengthen follow-up of patients*.*” (P06)*

## Discussion

This study explored both patient-related and healthcare system-related reasons for late diagnosis of breast cancer from patients’, family members’, and health care providers’ perspectives. Information on breast cancer risk factors, methods of early detection, initial symptoms, and its treatments are important for encouraging women to seek medical care immediately after recognition of the first symptoms [22, 23]. Our study, however, revealed a poor community awareness and common misperceptions about breast cancer, with little recognition of susceptibility or the benefits of early care seeking after symptom recognition. Because of poor knowledge about breast cancer, women are less likely to perceive the disease as severe and seek medical care if they are not acutely sick [24]. As a painless lump is the common initial presentation of breast cancer, many women disregarded the clinical importance of the first symptoms. Family responsibilities and inability to take time off from work increased the risk of disregarding the severity of the first symptoms. In accordance with these results, previous studies [25, 26] have demonstrated that women’s family engagements and job commitment are an important contributor to late/delayed medical consultation.

Moreover, it was rare to suspect cancer as a cause of the first symptoms of their breast illness. Most of the time the first symptoms of breast cancer were misinterpreted as resulting from non-cancer illnesses such as sun stroke, locally termed “mitch”. In Ethiopia, “mitch” is a local and common terminology used to explain a wide range of nonspecific illnesses in the community [27, 28]. Such beliefs triggered women to delay medical consultation while practicing different home remedies, spiritual acts and traditional medicines intended to treat the perceived cause of their illness, “mitch”. The misattribution of the symptoms is related to the women’s low perceived susceptibility to breast cancer, and poor awareness of the initial symptoms of breast cancer [29]. Similar to our findings, a previous quantitative study conducted in Addis Ababa found that more than half of women had poor knowledge about breast cancer [30]. Thus, increasing awareness of the signs and symptoms of breast cancer in the community could help to minimize such negligence and misattribution of the first symptoms of breast cancer.

Contrary to the misinterpretations of the symptoms, an initial suspicion of cancer deters women from seeking medical care immediately after they recognize the symptoms. This is mainly due to the view in the community that cancer is a deadly disease, and incurable with the existing conventional treatment in the country. Similar perceptions were noted in other studies from Ethiopia [16, 31], India [23], and Iran [26], and studies have reported women refraining from visiting health facility for fear of a confirmed cancer diagnosis [26, 31, 32]. This finding suggests that educating the community about the benefits of early diagnosis and treatment initiation could help to correct the misperceptions and improve health-seeking behavior of women with symptoms suggestive of cancer [33].

Previous studies in Ethiopia have documented that a significant number of people prefer visiting traditional healers before seeking medical care [24, 34]. Similarly, our study revealed that use of traditional medicine was common and the main cause of late diagnosis. For some of the breast cancer patients, seeking medical care was their second option, and only after traditional medicine failed to provide hope of a cure. Such practices have also been noted in other studies [34–36]. Together with late recognition of the first symptoms, the time elapsed on using traditional medicine could further advance stage of the disease and complicate its treatment outcomes. Therefore, involvement and working with traditional healers to create awareness about the symptoms of breast cancer and benefit of early diagnosis could benefit the women who visited the traditional healers for the complaint of breast abnormality.

The affordability of medical care [24] and quality of the health service [23, 37] determine the health care utilization of women who decide to seek care. Shortcomings in either of these could affect a woman’s decision to seek care from the health facilities or push them to seek care from traditional healers [31]. In our study, health system-related factors such as access to healthcare, physicians misunderstanding of the first symptoms, inappropriate reassurance of lump as a benign disease without biopsy, and poor counseling of patients presenting with signs and symptoms suggestive of breast cancer were noted as contributor to late diagnosis. Previous literatures have also revealed that health care providers’ failure to appraise the first symptoms of breast cancer appropriately, and misdiagnosis contributed to delayed diagnosis and disease advancement [26, 32, 38]. This may call the need for training primary health care providers [39, 40] to recognize the first symptoms of breast cancer, and increase the suspicion of breast cancer at first contact. Moreover, breast cancer patients need appropriate advice and discussion on their problem, its consequences and treatment decisions [41]. Most of the breast cancer patients were not ready to accept the diagnosis of their disease as cancer. Often, women became emotionally disturbed at the time of confirmation because of their low suspicion of being diagnosed with breast cancer, perceived fatality of cancer, uncertainty with the effectiveness of the conventional treatment, and fear of losing their breast through surgical treatment.

In contrast to the above barriers, the persuasion by family members and friends was found to be a facilitator for patients to seek medical consultations after they recognized breast cancer symptoms and to make the decision to undergo surgical treatment. This finding has also been documented in a previous study conducted among breast cancer patients and proxies in the same study area (Tikur Anbessa Specialized Hospital, Addis Ababa, Ethiopia) [14].

Early detection is the primary strategy for preventing premature death from breast cancer. Hence, interventions targeted at alleviating the above modifiable barriers [23] must be established in Ethiopia to diagnosis breast cancer at earlier stages. In settings, like Ethiopia, with no breast cancer screening service, early detection is mainly dependent on woman’s knowledge on breast cancer [12], performing regular breast self-examination and immediate medical consultations after recognition of breast abnormalities [42, 43], and immediate health care providers’ referral for confirmation [44]. Our study participants also emphasized the importance of breast self-examination, early medical care seeking, awareness creation and cancer pathology center expansion to improve early detection. Moreover, the study participants emphasized the importance of using breast cancer survivors as community educators to enhance community awareness of breast cancer. Though the effectiveness of this approach has not been investigated in our country, engaging breast cancer survivors as breast health educators [45–47] has led to significant improvement in the knowledge and prevention of breast cancer morbidity and mortality.

Our study included different groups of participants, which helped us to triangulate our findings and examine the reasons of late diagnosis from different perspectives. We attempted to interview recently diagnosed patients, however, recall of past events with a foresight of experience may unconsciously make the stories of these women biased and inaccurate in explaining their experience.

## Conclusions

The reasons for the late diagnosis of breast cancer among women in Ethiopia are multi-factorial and include individual, cultural, and health system factors. Interventions targeting these factors could alleviate the misconceptions and knowledge gap about breast cancer in the community, and shorten waiting time between symptom recognition and diagnosis of breast cancer.

## Supporting information

S1 File In-depth interview guide. Interview guides for breast cancer patients, family members, and health care providers at Tikur Anbessa Specialized Hospital oncology clinic.
(PDF)

S2 File De-identified excerpts. Themes and sub-themes based on analysis of open-ended responses.
(PDF)
