# Supplementary material for: Perspectives of patients, family members, and health care providers on late diagnosis of breast cancer in Ethiopia: A qualitative study
Source: PLoS One. 2019 Aug 1;14(8):e0220769. doi: 10.1371/journal.pone.0220769 (PMC6675093; doi:10.1371/journal.pone.0220769)
Supplement: S1 File — Interview guides for breast cancer patients, family members, and health care providers at Tikur Anbessa Specialized Hospital oncology clinic. (PDF) [file pone.0220769.s001.pdf]

Interview guides for breast cancer patients, family members, and health care providers at Tikur Anbessa Specialized Hospital Oncology Clinic

Annex I: English version Informed Consent form

Introduction: Thank you for agreeing to speak with me today. My name is ____________________________from in Addis Ababa University, School of Public Health. I come here to interview breast cancer patients. Breast cancer is the leading cause of morbidity and mortality among Ethiopian women. Early identification and treatment are the main interventions of premature death from breast cancer.

Purpose of the research: Despite the disease is the most common cancer in Ethiopian women, patients present to health facility very late after the disease gets advanced. However, very little is known about the causes of their delay. We are conducting this study with the aim of exploring the barriers to early diagnosis of breast cancer patients. In this endeavor we want to learn from your experience as to how you managed your illness and barriers to early diagnosis so that it will help us to learn about the barriers and further can help to design interventions to improve early detection of breast cancer in Ethiopia.

Participation: I am asking you and others to voluntarily participate in this study because you have been diagnosed to have breast cancer.

Risks: The study has no any risk for the participants and interview will be private to make safe participants from any fear. It is completely interview based; it has no any invasive procedure.

Benefits: You will not gain any direct benefit for being participated. But your participation is very important for us to learn about the barriers and find the answer to the research question which in turn benefits the society especially women.

Confidentiality: Any information that I collect about you during this interview will be kept confidential. Information about your identity will be put away after re-coding your file; and kept in a secured place. Only I will be able to link your identity with the code number.

Right to refuse or withdraw: You do not have to take part in this research if you do not wish to do so and refusing to participate will not affect your treatment at this hospital in any way. You will still have all the benefits that you would otherwise have at this clinic. You may stop participating in the research at any time that you wish without losing any of your rights as a patient here. Your treatment at this clinic will not be affected in any way.

Consent form

I, the undersigned, confirm that, as I give consent to participate in the study, it is with a clear understanding of the objectives and conditions of the study and with recognition of my right to withdraw from the study if I change my mind.

I ____ do herby give consent to ______________ to be interviewed. I have been given the necessary information about the research. I have also been assured that I can withdraw my consent at any time without penalty or loss of benefits. The proposal has been explained to me in the language I understand.

Patient's signature: _____________________________Date: _________/______/_______

Name of interviewer: Mr.: ______________________Date: _____/_____ / _____

Thank you for agreeing to participate

The interview will take less than an hour. I would like to ask your permission for taping the session because I do not want to miss any of your ideas and suggestions. Although I am going to take some notes during the session, I cannot possibly write fast enough to get it all down. Because we are on tape, please be sure to speak up so that we do not miss your ideas. I want to note again that the information you provided will be confidential. Any information we use from your interview will be combined with information from other women with breast cancer. It will not be possible to identify what you have said.

Do you have any questions before we start the interview?

Participant profile

**Age: ___________**
**Marital status: ___**
**level of education: ______**
**Occupation: _____________**

Annex II: Interview guides for women with breast cancer

1. What is known about breast cancer in the community?

   Probe:

   - causes
   - symptoms
   - screening
   - treatments
   - Support

2. Have you ever heard of breast cancer before you recognized the first symptom of your illness? (when, how)

3. Tell me about your experience with breast cancer from the first symptom recognition to diagnosis

Probe:

- Who noticed the problem with your breast?
- How was noticed the problem with your breast?
- What do you feel when you notice it?
- What action was taken after the problem was known? (before and after diagnosis)
- When do you seek medical care?
- Why do you seek medical care? (appearance of symptoms/persistence of symptoms/worsening of symptoms)
- When you were told you had breast cancer?
- Who was the first person you told about your diagnosis?
- What stage was the cancer diagnosed?
- Tell me about activities that helped you during your diagnosis?

4. What are the major challenges you have faced during diagnosis?

5. Why do breast cancer patients seek medical care late? (What affected that)

Probe:

- Socioeconomic factors (age, education, income)
- Individual factors (do not know where to go, carelessness/neglect, fear, financial, underestimate the severity of the problem)
- Cultural factors (traditional medicine, community beliefs)
- Health care system (access, referral)

6. What measures do you suggest to make sure breast cancer diagnosis happens early?

Probe:

- At individual level
- Community level
- Health facility level
- Government level
- What advice do you have for other women about breast cancer screening?

7. Do you have any questions for us or something you want to add?

Thanks for your time and information, have a good day!!!

Annex III. Interview guide for family members of breast cancer patients

1. Can you tell me about yourself?
2. What is the relation with patient?
3. Can we discuss on cancer?

   Probe:

   - What do you know about cancer?
   - How do people perceive cancer?
   - What about breast cancer?
   - Would you please tell us the local name given to cancer, if any!
   - What is the communities' perception about breast cancer? (causes, treatment, survival)

4. Can you explain me about your relative's (Patient Name) illness?
5. What was the feeling of your relative (Patient's Name) and yours when the illness was confirmed as cancer?

   Probe:

   - Who told the patient as her problem is cancer?
   - What was her immediate reaction?

6. How would your relative (Patient name) with cancer think about her disease?

   Probe:

   - for luck of hope,
   - fear of stigmatization, sexuality,
   - effect on marital status including possibility of divorce
   - how simple is to talk about cancer with the cancer patients

7.What are the major challenges your relative (Patient name) have faced during diagnosis?

8. Why the clients are not seeking medical care earlier? (What affected that)

Probe:

- Socioeconomic factors (age, education, income)
- Individual factors (do not know where to go, carelessness/neglect, fear, financial, underestimate the severity of the problem)
- Cultural factors (traditional medicine, community beliefs)
- Health care system (access, referral)

9. What measures do you suggest to make sure breast cancer diagnosis happens early?

10. Do you have any questions for us or something you want to add?

Thanks for your time and information, have a good day!!!

Annex IV. Interview guide for health care providers on cancer

1. Before we start our discussion, can you tell me about yourself?

   Probe:

   - Educational background, service year, working department

2. How long have you been involved in caring or treating cancer patients?

   Probe:

   - Type of cancer cases you are mainly caring
   - Your main role in the department

3. Can we discuss on cancer?

   Probe:

   - What do you know about cancer?
   - How do people perceive cancer?
   - What about breast cancer?
   - Would you please tell us the local name given to the cancer, if any?
   - What is the communities' perception about breast? (causes, treatment, survival)

4. How would patients with cancer think about the disease?

   Probe:

   - How do you communicate them about their cancer status?
   - To whom do you communicate first
   - How simple is to talk about cancer with the cancer patients?
   - How do you see the patients in terms of lack of hope, fear of stigmatization, sexuality?

5. Would you please tell us if there are issues related to disclosure of the status of a woman with breast cancer?
6. What could be the possible barriers for women to early diagnosis?
7. Do you have any questions for us or something you want to add?

Thank you very much for your time!
