# Supplementary material for: Perspectives of patients, family members, and health care providers on late diagnosis of breast cancer in Ethiopia: A qualitative study
Source: PLoS One. 2019 Aug 1;14(8):e0220769. doi: 10.1371/journal.pone.0220769 (PMC6675093; doi:10.1371/journal.pone.0220769)
Supplement: S2 File — Themes and sub-themes based on analysis of open-ended responses. (PDF) [file pone.0220769.s002.pdf]

Table 1: Themes and sub-themes based on analysis of open-ended responses

	Themes	*Excerpts*
Reasons for late diagnosis of breast cancer	*Lack of awareness about breast cancer in the community*	*"They (community) do not know it (breast cancer). We are confused with the cause of the disease." (P01)*<br><br>*"I have never heard any discussion about breast cancer in the community. Rather they say, I get pain in my breast, it is something like cold…" (P08)*<br><br>*"They said there is a method. But I do not know it. There are people who made check-up." (P02)*<br><br>*"It is easier to say that the people have no awareness or have wrong information on cancer when compared to the medical related knowledge and other countries experience with the same age group and similar part of community. They have very limited or wrong information about cancer. Very small parts of the community, like 10% of them have a better awareness on the disease. Especially educated and people from urban area have better knowledge about cancer where attendants have more knowledge than the patients. It is easier to say that other parts of the community have no awareness or have wrong information about cancer. Some of them think the disease is deadly or have no treatment and some of them think that it is just like any other disease where they take some medication and get back to their normal life after being cures. Both are wrong information because they perceive that it is very deadly or as easy as having an operation. But some of them have good information and ask relevant questions and some of them ask questions completely unrelated to the disease." (HP04)*<br><br>*"It is very tough. All the people do not have a good perspective towards it. Their approach is difficult. They ask you from where or how you got the disease. But I do not know anything about that…People do not like it when you told them that you are sick." (P10)*<br><br>*"I picture it as something huge and tough…Even though I do not know the details; I heard that it has no cure. Moreover, I heard that many people are dying because of it. That is how I understand it…From what I observed on my mother it does not give you time or it progresses fast. …I told you that they do not*

		have the awareness. Nobody created awareness among the community. They just know that it is fatal. They perceive that it make the patient suffer before it kills him/her. They do think that it just kills you immediately and do not have any positive thinking towards it." (R04)<br><br>*"Even though the disease existed before, I do not think people had awareness about it. Moreover, people were not told that they have cancer in the previous days. They just follow up the treatment whether it is chemo or other therapies but they were not usually told about the diagnosis… I do not think people in my surrounding have the awareness." (R03)*
	Disregarding or misattribution of breast cancer symptoms	**"***I noticed a small swelling. But I was not worried with it. The swelling was very small. I ignore it. I took it simple. I have never thought that will lead me to this much pain. But latter I started to feel pain. Latter, it starts to extend its tail to this (towards her arm pit). Then I went to the health center. They told me to go to the hospital. I did not thrust them. I did not consider as serious. I considered the disease nothing. When I get severe pain, I returned back. The doctor gets angry with me." (P01***)**<br><br>*"I felt the swelling in my breast. I felt it accidentally when I was changing my cloth…. It could be negligence. Most of the time cancer is painless. So it does not make you to be worried when you do not feel pain."(P02)*<br><br>*"When I was dressing my pajamas, I noticed something small hard thing on my breast." (P07)*<br><br>*" … I bought and apply new breast bras. Then I feel pain in my breast after three days. I fill itching and then it gets swollen. Then I applied a holy water. Then I got better. It disappeared. Then it returned back again." (P03)*<br><br>*"I started to feel itching sensation on my breast. It is intermittent itching. I have no pain. It is only itching. …" (P08)*<br><br>*"Then I did not do anything about it; I hidden the disease…I taught it was a simple thing so I did not tell anyone … I went to a traditional healer…I did not tell anyone. I went to the traditional healer by myself.*

		The traditional healer gave me an ointment, which I used for 3 or 4 months. Then there was no change with the swelling…It wasn't wounded but it was very painful to touch. There was no problem except for that. The traditional healer tried to give me another medication but I refused and told him that he is not helping me and I wanted to go to the doctors. After that, I talked to some people and they told me to go to the health facility if it is a breast related case. Too long … For more than a year. It had no pain. I had severe burning sensation. …" (P12)<br><br>*"I have never thought it will be cancer. We have no such history in our family. My illness was intermittent. Come and cease for a month and more… for the last one year, I did not give any attention. I had no pain during that time." (P06)*
	Misperceptions about breast cancer treatment and its outcomes	*"Sure mine is also caused by mitch* (sun stroke)*. It is caused by prolonged exposure to sun with the exposure of your body while you have sweating to sun light. I work in the street. It is caused when I takeoff my t-shert while I felt hot in the day time." (P02)*<br><br>*"It is not unusual to say I am going to die. Because the people say cancer is bad disease. Then I said God know everything. It is the will of God." (P03)*<br><br>*"… It is God's will. I do not know what I missed. God had given me this disease and I received it. Even now, it is the wills of God to be cured… yes; I scared when its name is called. It has no treatment. I did not still get complete solution to my disease. It cannot be cured. I am still suffering." (P05)*<br><br>*"I do not know it. I felt as God exposed me to this disease… What I thought and which is always in my mind is; I used five year implanon contraceptive in my arm. I suspect it could be the cause. I do not know it because I could not get the cause of my problem."* **(***P07***)**<br><br>*"I have never even had a headache except this disease. I had this disease recently. It is sent from God so it is better to accept it…They told me that I will be cured and I did. I am still on follow up thanks to God. I am taking a medication and feeling better now … How are they going to forget about the disease; they are carrying it…They think that they might die…They think that the disease cannot be cured and the*

		person who has it is just waiting to die. This is what they say…" (P09)<br><br>*"I wash after cooking meal but do not know how it is caused. You never know the heat and the odor might cause it since we fry food and go outside…"We can't say anything except asking God to end our suffering after this…They are very unhappy and worried…They are worried because of the diseases." (P10)*<br><br>*"I did not know the cause of the disease… The community says something. I heard a woman saying that she had the disease because of evil eye." (P12)*<br><br>*"…She is fine now. God knows about the future…She might have a hope to be cured. She is fine…" (R02)*<br><br>*"Didn't I tell you that people think cancer as a fatal disease …That was how she felt. She tried to give us everything and did not even think about herself at all. She said that she do not have life after the incidence." (R03)*<br><br>*"Yes, there is. Our society is very religious due to this some of the patients ask God why he brought the cancer up on them. It might be because their perception about the disease severity or things they heard, they ask why they have such awful disease. They feel like they did sinful thing to deserve such disease. Some of the patients relate the disease with the things they eat and drink or smoke. These are usually common in breast cancer patients… Most of them do not know the exact cause of the disease…" (HP03)*<br><br>*"Most people fight to survive even patients with last stage of the disease feel like they will get cured if they are treated here." (HP04)*<br><br>*"It is confusing. But most of the educate people distrust, I think this is because they read about it. Uneducated people also believe that they will not get cured because of the information they got from their society. There are also people who hope to get cured related with their religious believe." (HP01)*

		"Even though I have never interviewed the clients formally, from my understanding I think there are two types of perceptions. Educated people and people from the urban area know about the disease but they only know about the severity of the disease. They do not listen to most of the things we had to say and have lack of perceptions about the treatment because they already gave up hope on the outcomes." (HP03)<br><br>*"People who are coming from rural area and don't have any clue about cancer, will believe as they will get healed after they receive the medication. Therefore, they immediately ask you," so what shall I do then? What is the next step?...Educated people get really shocked since they have some exposure about cancer and all of them assume as they will get died after a while. Most of these types of people want to know more about their status, survival, how much the treatment will benefit them and so on. They just want to know more about their situation in detail." (HP05)*
	Non-medical management of breast cancer symptoms	*"They believe on holy water. It is the will of God. Even a doctor cannot be succeeded if God does not help him. Everything is under God. There are women who cured by holy water. The other is traditional medicine; for instance one woman who comes to visit me, told me that her breast was burst. Then she went to traditional healer (name), and cured. She showed me three healed scars on her breast. I witnessed it, she get cured. …"(P06)*<br><br>*"They use hot remedies and apply a leaf of mitch. We perceive as we catch mitch while we work in the field, then we squeeze the leaf and apply on it (the breast)." (P01)*<br><br>*"I heard that there are women who cured by holly water. While I was in the hospital the people coming to see their relatives said me why don't go to holy place. There are people who completely healed by holly water…I heard about the presence of traditional medicine but I do not know where it is. I said them I do not want to go there. While I was in the hospital, one of my relative come and said me you should not cut your breast, we should go to the traditional healers (Name). Then I told him I will not try any traditional medicine, the doctor will do whatever he decided. Therefore, there are hearsays about the presence of traditional medicines. There might be women who went there". (P06)*

		"I put some holy water and Emnet (holy soil) on it the first day I got sick then I got cured. I went to work after that. Then it started again after 5-7 days." (P10)<br><br>*"There are people who say there is traditional medicine even in Addis. …they insert something using syringe. Then they said that medicine will get out the cancer from the breast. The inserted medicine will pull out the cancer... The people are scared with the word cancer. They go to holy water. Many people go to holy places. They said God know for us. It is due to the fear of the cancer. For example I went immediately to holly place. First I visited the health center. They write me a letter of referral to the biopsy place. My mind was disturbed during that time. I left the referral paper and immediately went to holy place. Then I get stable with my mind after I washed the holy water in St. Tsadikan Marriem." (P02)*<br><br>*"I felt pain and got the swelling. In our village we usually think mitch (sun stroke) if we feel ill. I tried remedies for mitch. Then I start to feel more pain, chills, started to loss my body, and lost appetite. Then I went to holy place and washed holly water for a long time. I used holy water for about a year thinking I will get better by the holy water." (P05)*<br><br>*"There are some people who will totally give up and refuse to take the treatment. They prefer to go to other traditional places/ holy water. Then, they will return back to you after a year in which the cancer is already advanced and complicated." (HP05)*
	Fear of cancer diagnosis	*"There was a discussion about cancer on Television. But I was not hearing it because I feared its name and I was thinking could this happen to me. It happened to me, my fear become real. I was going out when the television starts to talk about cancer …Then he told me your problem is cancer ... Then I cried day and night saying why this happened to me." (P07)*<br><br>*"… When I tell to my sister, she told me to visit health facility. She said it is not good to wait. I get scared. … I do not know. I perceive as it will kill me immediately if it is cancer. Then I feared going to the health facility… I do not know. I perceive as it will kill me immediately if it is cancer. Then I feared going to the health facility. My uncle come and advised me. …In the community it is said that you cannot cured*

		from cancer. It is called a killer. I lost my hope… I do not know. I perceive as it will kill me immediately if it is cancer. Then I feared going to the health facility. … In the community it is said that you cannot cured from cancer. It is called a killer. I lost my hope." (P02)<br><br>*"I cried. I lost my hope. … I scared when its name is called. It has no treatment. I did not still get complete solution to my disease. It cannot be cured. I am still suffering … the doctor told me, you will be operated … But I hesitated saying how I can do this? Who will take care of my children? I denied saying I should feed my child and make them grew. I got them with a lot of promise to God. What if I die while I was operated … I feared the operation. I denied them and returned to my home. I feared when the physician told me half part will be removed. I completely leave it and stayed for around one year in my home." (P05)*<br><br>*"…They (physicians) told me it is tumor, but it is poisoned tumor (it can be distributed to the rest of your body). He told me we are going to remove completely. When he said it, I shocked and cried. The people with me get disturbed. Then the doctors discuss and wait me for more four days until I get calm down and stable. Then it gets removed…" (P06)*<br><br>*"Oh, my God, I felt many things, having a breast removal at old age ... I had no pain at all. I felt why I have to go through breast removal at this age. I should die and get buried with dignity. I felt very sad." (P13)*<br><br>*"Moreover, they say that it is shameful if someone hears about it… A person should die with full body parts, but part of the body will be lost due to the breast removal" (R03)*<br><br>*"… He (physician) told me we are going to remove completely. When he said it, I shocked and cried. The people who were with me get disturbed. Then the doctors discuss and wait me for more four days until I get calm down and stable. …" (P06)*<br><br>*"...I think anyone would feel that way… Anyone will be shocked, except in rare cases. You might even die of shock ..." (R01)*

	Competing priorities	*"It is simply saying our home will be affected when we go out. Because we have children who should go to the school, we need to prepare food. There is additional Kebele (smallest administration unit) developmental works. We have a lot of works. Therefore, it is a problem. You cannot leave them and run to the health facility. You cannot leave your home alone." (P05)*<br>*"I stayed saying I will get better. I had children. I should work to serve my children. I was alone. There was no one in the home who could serve my children…" (P08)*
	Financial insecurity	*"… I went to the health center. They told me to go to the hospital. However, I had problem of money and returned to my home and stayed for a month … then later I went again to the health center. He (Health professional) strictly told me to go to the hospital immediately in that day. He paid a transport cost for me." (P01)*<br><br>*"Yes, where they can get money. We have shortage of money. We need to sell our cattle or our crops to get money otherwise we had nothing." (P05)*<br><br>*"There is transportation cost, medication cost. They may get it difficult to get these. Our people are poor. The people are not educated …" (P07)*<br><br>*"... It is difficult to get treatment from private health facilities. It costs more than 2000 birr to get the treatment and buy medications. If you visit private health facilities it might cost you from 20,000-40,000 birr. You cannot just come here (public hospital) and get the treatment; you have to get permission from the village administrators first. You have to settle some of the things first, you cannot get the treatment easily" (P03)*<br><br>*"I asked them how much I have to pay to check its price, if it was expensive. I thought it was cheap but it costs 7500birr. They told me the sample would be sent to German thus I collected the money from different place and paid for the diagnosis because I did not want her (his wife) to die." (R01)*

	Health system-related barriers	*"... I noticed something small hard thing on my breast. ... I get worried; I stayed the night thinking what it could be. There was a doctor in our neighborhood. I wake up in the morning and showed him. He told me it could be tumor. I said him if it could be a cancer because I heard on TV. He said me it is simple; it is not cancer." (P07)*<br><br>*"... The doctor immediately advised me to go to X Hospital. When I went to X hospital, they examined me and told me half part of the breast will be removed... Yes, I feared the operation. I denied them and returned to my home. I feared when the physician told me half part (Breast) will be removed. I completely leave it and stayed for around one year in my home. Then my family relatives told me that there is a new doctor who is special to do the operation. I decided to go and thanks to God and the doctor. He is a blessed doctor..." (P05)*<br><br>*"They told (diagnosis) to my husband. He (doctor) did not tell me. .... Then he told me your problem is cancer, calm down, you should not wait, it should be removed before getting disseminates to your body and we can't touch it if it is disseminated. Then I cried day and night saying why this happened to me ... It (hospital) had no all instruments. I said, how can I have operated without full investigation made and specified my problem? They have no instruments for testing biopsy (namuna)." (P07)*<br><br>*"You go to the health facility immediately if you have flu but this disease needs a lot of investigations. They will give you syrup and tablet immediately but this disease needs admission and a continuous follow up. It needs a lot of work to have a registration card, see the doctors, and find a bed for admission and the like. For example, I handled all this alone even if I was sick." (P02)*<br><br>*"...Yes it was not because the disease did not exist. From what I understand the disease is not easily diagnosed during investigation. ...Do you mean that the doctors could not identify it easily? ... Yes, that is what I understand ...I do not know why." (R04)*<br><br>*"There is a radio and chemo therapy. The women I was taking care of had chemotherapy from private facility but we are still waiting radiotherapy. The provider told me the machine is not functional. It has*

		been six months since we register and the women came from the region. Can you help us on that?" (R02)<br><br>*"I understand that it is a sever/bad disease. Most people come to the facility after the disease progress and there is a scarce treatment for everyone and also long waiting time to get treatment" (HP01)*<br><br>*"For example, most of them go to religious places for holy water before starting the treatment if they have breast cancer and come back at the advanced stage of the disease (4th stage). It is difficult to treat the case at that stage because it is already disseminated." (HP02)*<br><br>*"The problem with that is there is a long waiting period to get treatment. Due to this reason, patients have less hope. …We face this challenge especially with 3rd and 4th stage cancer patients. They might have wound and discharge with bad odor since we cannot treat them immediately due to long waiting period."(HP03)*<br><br>*"To be frank we do not have the time to discuss with the patients about the disease… It is difficult to give time for outpatients in this situation… Sometimes we just use question and answer approach. We just ask the symptoms and tell them the treatment options …" (HP04).*
Facilitators of early diagnosis of breast cancer	*Persuasion by family members and friends*	*"I do not know. I perceive as it will kill me immediately if it is cancer. Then I feared going to the health facility. My aunt come and advised me. She told me lets know what type of problem is this; it may not be what you feared for."(P02)*<br><br>*"The retraction of my nipple and the influence of my friend make me seek medical care. She worried me saying why do you give time, it is a breast. Had she did not pushed me, I might not seek care. …" (P06)*
	Prior knowledge of someone with breast cancer	*"… I was planning to go to the holy water. But I saw a mother who was treated for breast cancer. Then I shocked and run to the hospital. I did not face any illness. I visited hospital immediately." (P04)*
	Literacy level of the women	*"The young are better. The old women did know nothing. Like me." (P08)*

		"Those who are educated are more likely to seek care earlier. Had I were educated, I seek medical care earlier. It is due to lack of knowledge which makes me to stay this much time. Otherwise I wish I could visit immediately had I have been educated…" (P05)
Participants' suggestions to mitigate late diagnosis		*"The women should assess their breast always…"(P03)*<br><br>*"While they are taking shower, … Especially breast and womb is very serious. ... So mothers should assess their body and follow their body … they should assess their breast always; they should visit health facility and seek advice from a doctor when they feel something. It is what happened for me. It is because I stayed silent and resulted to this stage." (P06)*<br><br>*"They should not wait for a moment after they notice, they should not be negligent." (P02)*<br><br>*"Now the radio is talking about the severity of cancer. It is telling the community about the problem. The community is getting conscious on the disease. Now it is cancer which is getting the highest concern as it was HIV in the previous time. So, it is helping them to seek medical care earlier… the government should extend the service; support the poor to get the service and education to the community." (P01)*<br><br>*"We are saying that the disease is spreading or affecting many women however, most of the services are in Tikur Anbessa Specialized hospital. Therefore, there should be more facilities which should give similar services. The government should open more facilities providing these services and strengthen the follow-up of the mothers."(P06)*<br><br>*"I for example, I am teaching the community. I am telling them as they should seek medical care immediately. You should not stay at home. I tell them it can be changed to other diseases if you did not seek care immediately." P05)*<br><br>*"… They should come earlier. If they ask me about where they should go, I am happy. I can tell them. I can assist them." (P08)*

	" ... You should educate the community that cancer can be cured and should go to the health facility as soon as they have the symptoms. For example, if I had known about it, I might have gone to the facility earlier or before it got worse…It is better to go house to house and teach the women about the problem… We were thought about HIV…cancer is not different from that. To be frank many people are getting sick thus, education should be continuous… It is better to educate them while gathered…You can do it at each surrounding. It can be done under every health center but not hospital. It might not be possible to gather the patients at a certain place but you can educate the patients who are already here. You can discuss the issue while patients are waiting for their turn. I think that is important… About the disease, causes, and symptoms and to visit health facility immediately and the like… I want to know about it before I got the disease but I do not know about other people perspective on this matter. People do not think the same way when they face it and before that. Thus, there is nothing like knowing about it before it happens… We do not have any barrier. Knowing about it and saving your family member is not an easy thing. I am ready to teach the community if I had the time and if I am feeling better. This is my perspective and I do not think anyone will disagree to save the lives of others" (P03)<br><br>*"I do not have anything else. If you create awareness, the people will have more understanding as well us knowledge." (R04)*<br><br>*"I am not educated much. It is good if the women visit health facility before the disease got worse. It will be cured if they seek care early. However, it is difficult to be cured after the disease progress. That is how I understand it."(R05)*<br><br>*"No, it might be cured. If the disease is treated early and before it got worse, it will be cured. I have seen people being cured. However, if it is treated after it already caused damage; the treatment will only help for some time. It does not have much effect." (R02)*<br><br>*"What I want to say is that the main discussion point should be to stop the disease progression by early diagnosis and treatment. Hence, it would make me happy if we work on the prevention. It might be*

	difficult to prevent the disease completely but it is possible to delay the progression." (R03)<br><br>*"I think it is a bad disease but if they come early they will be cured. That is what I know. They have a better chance if they come here, that is the information I have. But they usually come at the last stage of the disease so they go through only palliative care." (HP01)*<br><br>*"They will tell you that you have the disease after they confirm it right? Thus, the doctor should be friendly and should not upset the patient. Even other patients want politeness let alone cancer patients. The doctor should be polite, advice the patient not to be upset and to be clean. Even though patients might do this by themselves, it would be better if they get counseling from the doctors. I am saying this based on my experience related with my mother. My mother's doctor is called Doctor X and my mam feels like he (doctor) is the one who healed her. She will tell you that God saved her if you ask her. He is very polite." (R05)*<br><br>*"There might be poor patients who might not even have money for transport. Thus, it is better to find a way for those patients. Even though you just want to conduct the study, there are people who comes from rural area and do not have a place to stay. It would be better if you could support them." (R04)*<br><br>*"I think it is better to tell them … They will be cautious. It is possible to prevent the disease since there is a technology to inform people….Yes that is true they will be shocked. But what you have to think about is the next step after they are shocked. If they know about the disease, they will go treatment early and take their children for checkup….Even though it is difficult, it is better to talk about it so that people have awareness." (R03)*
